# Moral Suasion and the Private Provision of Public Goods: Evidence from the COVID-19 Pandemic

**DOI:** 10.1007/s10640-020-00477-2

**Published:** 2020-08-17

**Authors:** Björn Bos, Moritz A. Drupp, Jasper N. Meya, Martin F. Quaas

**Affiliations:** 1 Department of Economics, University of Hamburg, Von-Melle-Park 5, 20146 Hamburg, Germany; 2 CESifo, Poschingerstr. 5, 81679 Munich, Germany; 3 Department of Economics, Leipzig University, Grimmaische Str. 12, 04109 Leipzig, Germany; 4 German Centre for Integrative Biodiversity Research (iDiv) Halle-Jena-Leipzig, Deutscher Platz 5e, 04103 Leipzig, Germany

**Keywords:** COVID-19, Coronavirus, Public good contributions, Moral appeal, Moral suasion, C93, D62, D64, H41, I18, Q58

## Abstract

We study how moral suasion that appeals to two major ethical theories, Consequentialism and Deontology, affects individual intentions to contribute to a public good. We use the COVID-19 pandemic as an exemplary case where there is a large gap between private and social costs and where moral suasion has been widely used as a policy instrument. Based on a survey experiment with a representative sample of around 3500 Germans at the beginning of the pandemic, we study how moral appeals affect contributions with low and high opportunity costs, hand washing and social distancing, to reduce the infection externality as well as the support for governmental regulation. We find that Deontological moral suasion, appealing to individual moral duty, is effective in increasing planned social distancing and hand-washing, while a Consequentialist appeal only increases planned hand-washing. Both appeals increase support for governmental regulation. Exploring heterogeneous treatment effects reveals that younger respondents are more susceptible to Deontological appeals. Our results highlight the potential of moral appeals to induce intended private contributions to a public good or the reduction of externalities, which can help to overcome collective action problems for a range of environmental issues.

I firmly believe that we will succeed in this task if all citizens truly see it as THEIR, as YOUR task. [...] The action of each individual counts. We are not doomed to passively accept the spread of the virus. We have a remedy: we have to keep a distance from each other out of respect for one another.

(Chancellor Angela Merkel, March 18, 2020)

## Introduction

Moral suasion is a prominent instrument for aligning individual and public interests (Romans 1966), and one that is increasingly entering the field of environmental economics (e.g. Carlsson et al. 2019; Ito et al. 2018). As a non-pecuniary instrument, moral appeals are outside standard economic cost-benefit analysis. Their monetary costs are typically small, they are fast to implement, and they can complement economic incentives or command-and-control regulations. By affecting social norms or the adherence to norms (Nyborg et al. 2016; Young 2015), moral suasion is expected to increase individual contributions to reduce externalities or to contribute to a public good in absence of more rigorous governmental interventions and may also increase support for and compliance with regulations. Although a number of studies examine the effect of moral suasion on the compliance with already existing legal rules,^1^ there is little knowledge from the field to what degree this extends to voluntary private public good contributions. Carlsson et al. (2019) review the literature on nudging as an environmental policy instrument and identify four studies using normative appeals: Goldstein et al. (2008), Egebark and Ekström (2016), Kallbekken and Sælen (2013), and Ito et al. (2018).^2^ All these studies use rather general normative appeals and do not explicitly draw on a specific moral theory or school of ethics. Thus, scrutinizing the effect of specific moral appeals on voluntary public good contributions is an important research gap.

To contribute towards filling this gap, we examine the effect of moral appeals—according to Consequentialist and Deontological ethics—on intended contributions to a public good drawing on a large, representative survey experiment with around 3500 Germans. We use the COVID-19 pandemic as an exemplary case in which private actions have substantial external effects, and in which moral suasion has been prominently used by heads of state—as exemplified by the translated excerpt of a very rare television address by German Chancellor Angela Merkel. Physical social distancing or increased hand-washing, for example, not only reduce the private risk of an infection but also reduce the risk of infecting others. Epidemiological-economic models show that there is a considerable gap between individually-chosen and socially-optimal behavior (e.g. Acemoglu et al. 2020; Farboodi et al. 2020; Gerlagh 2020; Quaas et al. 2020). That is, the social cost of contacts outweigh the private cost by orders of magnitude (Gerlagh 2020; Quaas et al. 2020), qualitatively akin to the case of the social cost of carbon. Thus, our analysis is not only of relevance for public health in the COVID-19 pandemic, but the insights may extend to a range of other applications where private actions have external effects—many of them at the heart of environmental economics. Prominent examples are Greta Thunberg’s and others’ moral appeals to increase the efforts of mitigating climate change.

Our study explores the effects of moral appeals in line with the two major theories of moral philosophy that normative economic analysis draws on—Consequentialist and Deontological ethics. According to Consequentialist ethics, moral evaluation of some action should be based on the expected outcome of that action. In contrast, Deontological ethics, with Immanuel Kant being one of the prime proponents, instead, focuses on the *duty* to do the morally right action, irrespective of outcomes. Traditionally, welfare economists have focused predominantly on Consequentialist ethics, with Utilitarianism as the consequential moral theory that has become most influential to economics (e.g. Mill 1859; Harsanyi 1953; Maskin 1978). Deontological ethics, instead, have gained attention in normative economics more recently (e.g. Roemer 2019).^3^ Apart from their relevance in the history of economic thought, both ethical approaches are present in public debates and German citizens are in principle familiar with both of them in their translations to everyday life.

To study how moral appeals in the spirit of these two schools of ethics impact planned contributions to slow the spread of the coronavirus, and thus contribute to public health, we conducted a pre-registered online survey experiment with 3616 Germans in March 2020. We randomly assigned respondents into two treatment groups and showed them moral appeals from a medical doctor who is treating COVID-19 patients. Respondents in the *Consequentialist* treatment saw a message highlighting the *consequences* of physical social distancing and washing hands for the health of others. Respondents in the *Deontological* treatment, instead, saw a message emphasizing the *duty* to act in a way that does not harm others and that could serve as a blueprint for the behavior of others. Finally, we compare responses about the planned defense efforts of respondents in these treatment groups to those of participants in a control group who did not see any moral appeal.

Consistent with our pre-registered hypotheses, we find that moral appeals can increase planned private public good contributions. The Deontological appeal has a particularly strong effect, increasing both planned high-cost and low-cost public good contributions, the reduction of contacts and the increase in hand cleaning effort, respectively. The Consequentialist appeal is only effective in increasing planned hand cleaning efforts. Both moral appeals trigger an improvement in support for and accordance with regulations relative to the control group. Finally, our analysis of heterogeneous treatment effects suggests that appeals are particularly effective for younger individuals and pronounce existing altruistic preferences.

Our paper adds to the literature on moral suasion, moral preferences and private public good contributions (e.g. Andreoni 1990, 2007; Bénabou et al. 2018; Dal Bó and Dal Bó 2014; Daube and Ulph 2016). With regard to moral appeals in the context of public good contributions, Dal Bó and Dal Bó (2014) show that moral appeals in the spirit of Utilitarianism and the Kantian Golden Rule can increase public good contributions in the lab. Closest to our focus of examining intended contributions to a public good in the field is the pre-print by Everett et al. (2020). Five days prior to our study, they independently conducted a survey on public health behavioral intentions in the COVID-19 pandemic with around 1000 US participants and show them Facebook statements from a high school teacher or a director of the Department of Education. Besides a Consequentialist and a Deontological appeal, they also included a Virtue appeal. However, they do not find significant effects of moral appeals on physical social distancing. This could be due to various factors, such as their smaller sample size, cross-cultural differences or the profession of the person that appeals to participants’ morality. In comparison, our contribution rests on a much larger sample size from a country where Deontological ethical positions are likely more prevalent as compared to the USA.

The remainder of the paper is structured as follows. In Sect. 2, we describe our experimental design. We present our results in Sect. 3 and conclude in Sect. 4.

## Experimental Design

**Fig. 1:**
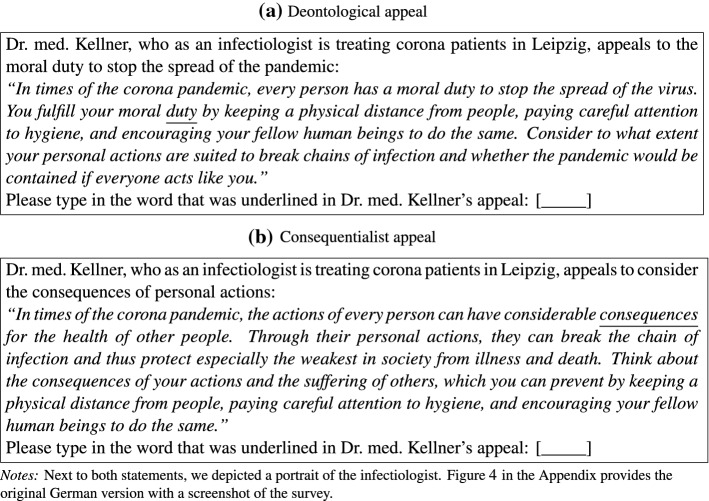
Moral appeals

We conducted an online survey experiment with 3616 Germans from March 20 to 27, 2020.^4^ In the first part, participants answered questions about sociodemographics, their risk-preferences, expectations about their risk-exposure, and their past behavioral changes. Afterwards, we randomly assigned them into one of the two treatment groups or the control group with equal probability.^5^ Participants in the *Deontological* [*Consequentialist*] treatment then saw a statement by a medical infectologist who is treating COVID-19 patients with a *Deontological* *[Consequentialist]* appeal to tackle the COVID-19 spread, whereas participants in the control group were not exposed to any message. Figure 1 depicts the exact wording of both statements. We chose to frame these statements in a way that conveys the broader sentiments of each school of ethics, instead of changing only a single word. Next to both statements, we depicted a portrait of the actual infectologist, to ensure credibility and to signal leadership, which has been shown to increase voluntary public good contributions (Dannenberg 2015). To ensure that participants carefully read the statement, we asked them to enter the underlined word in a text field below the statement as a treatment check.

After providing these moral appeal treatments we asked participants about their planned private public good contributions in the next week. In particular, we asked: “Compared to the same week last year, by what percentage will you reduce or increase your physical, social contacts in the coming week?” and “Compared to the same week last year, by what percentage will you reduce or increase your intensive handwashing (longer than 20 s) in the coming week?”. In the survey, we defined “physical, social contacts” as situations in which the respondent comes closer than two meters to others. For both questions, we collected responses on a 15-point log-scale ranging from “reduction to one-tenth” to “tenfold increase”. Both actions have a mixed private-public good character where the relative weight given to the private or public benefit can differ between both actions. The effect is different for those who have not yet been infected and for those who are infected and infectious. For the infected, the public good character of contact reductions is eminent, whereas the public good character of hand cleaning was made salient in the public debate due to uncertainty about potential transmission channels. Three weeks after our data collection, for example, van Doremalen et al. (2020) showed that SARS2-CoV-2 can stay viable in aerosols and on surfaces for many hours, such that transmissions can potentially happen regardless of contacts. Hence, hand cleaning effort is also an important contribution to public health. Also for the not yet infected (‘susceptible’) people the efforts to protect themselves from an infection provide a private, but also a public good. This is because they also protect themselves from becoming infectious and thus reduce the risk that they could eventually infect others. Given the large number of susceptible individuals, this is a quantitatively important contribution to the public good (Quaas et al. 2020).

In addition, we asked for the support for governmental actions and by how much participants reduce their contacts with regard to governmental regulations.^6^ In the “Appendix”, we provide a list with all relevant survey questions and their full range of answer options (Table 9), illustrate the distribution of answers per treatment group (Fig. 3), and provide descriptive statistics of relevant survey responses (Table 2). As participants have been anonymous, we cannot check how truthful single participants answered or whether their answers have been biased by social desirability concerns. On the aggregate level, however, we can check for a systematic difference between stated answers and observed mobility data. In a companion paper (Quaas et al. 2020), we examine the trend in cell phone movements in Germany and find a reduction in cell phone movements for that time period which is consistent with the survey answers. Hence, while we cannot rule this out, we do not expect such systematic biases in survey responses.

During our data collection, the German government announced a nation-wide contact ban on March 22, 2020. This regulation prohibited meetings with more than one other person at a time, except for household members, but did not constrain the total number of daily contacts. Quaas et al. (2020) show that the contact ban had no effect on the motivation for private public good contributions and increased support for governmental actions. As the contact ban was independent of our survey treatment, it affected respondents in control and treatment groups similarly, i.e. we measure the treatment effect independent of the ban.^7^

In line with the motivation outlined in the introduction, we expect the moral appeals to enhance the planned private public good contributions. More formally, we hypothesize:^8^

### **Hypothesis 1**

Deontological and Consequentialist moral appeals increase planned defence efforts, measured in terms of social distancing or hand-washing, as compared to the control group.

Moral appeals approach the respondents in their role as citizens. Hence, appeals that highlight the morally right behavior, could also increase the support for government actions that are meant to benefit the common good. Thus, we hypothesize:

### **Hypothesis 2**

Deontological and Consequentialist moral appeals increase support for governmental regulations or behavior in accordance with governmental rules as compared to the control group.

To test these hypotheses, we employ the following econometric specification:

$$\begin{aligned} y_i = \alpha + \beta _1 T_{1i} + \beta _2 T_{2i} + \gamma {\mathbf{X }}_i + \epsilon _i, \end{aligned}$$

where $y_i$ denotes, for respondent *i*, one of our four outcomes, i.e. the change in planned contacts, the change in planned hand cleaning effort, the support for governmental regulation and the change in contacts with respect to governmental regulations. The vector ${\mathbf{X }}_i$ captures respondent *i*’s age, gender, and dummy variables for their education (5 groups) and household income (4 groups), and $\epsilon _i$ is the error term for respondent *i*. With $T_{1i}$ [$T_{2i}$] indicating if a respondent is in the Deontological [Consequentialist] treatment, we estimate the treatment effect relative to the control group and test our hypotheses with $\beta _1$ [$\beta _2$].

## Results

### Main Results

**Fig. 2:**
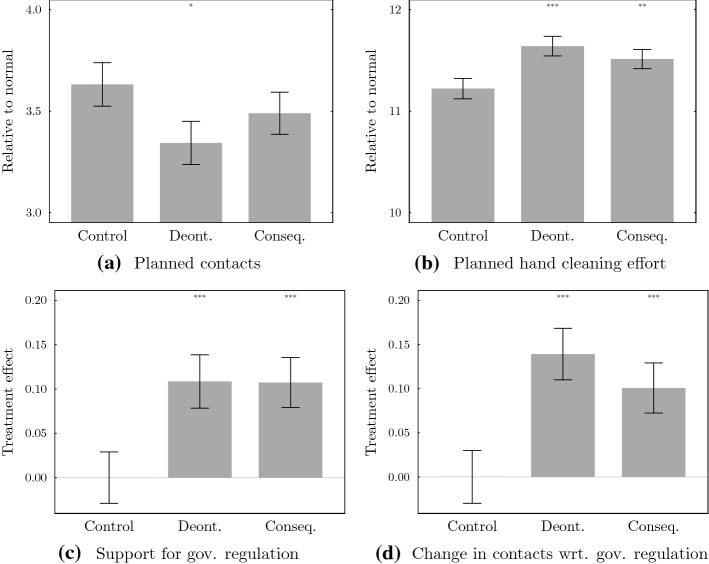
Average planned private public good contributions by treatment group and treatment effects on the support for governmental regulation. *Notes* Bars indicate mean values or treatment effects and error bars the respective standard errors per treatment group. In **a** and **b**, we depict the average level of contacts and hand cleaning effort compared to pre-pandemic normal levels from the previous year on a 15-point log-scale ranging from “halving” (3) to “reduction by 33%” (4) in **a**, and from “increase by 5%” (10) to “increase by 48%” (12) in **b**. In **c**, **d**, we depict the treatment effects on the support for governmental regulation and the change in contacts with respect to governmental regulation. For the latter two variables, we use their z-score to allow for an interpretation in units of standard deviations. Stars indicate the significance of differences compared to the control group (t-tests): *$p<0.1$, **$p<0.05$, ***$p<0.01$

**Fig. 3:**
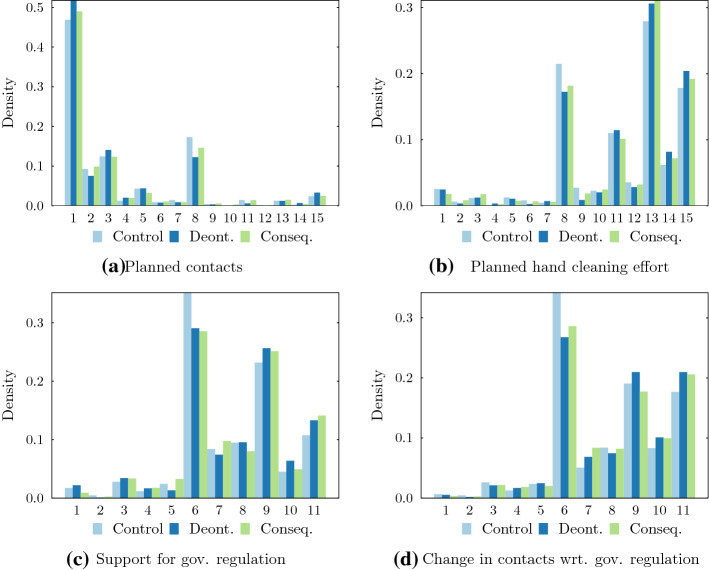
Distribution of outcome variables by treatment group. *Notes* The answer options for the planned number of contacts and the planned hand cleaning effort in the next week range from “(1) Reduction to 1/10” via “(8) No change” to “(15) tenfold increase”. The answer options for the governmental support range from “(1) are way too much” via “(6) are appropriate” to “(11) are way too little”. The answer options for the change in contacts range from “(1) Participation in coronaparties”, via “(6) according to regulations” to “(11) complete stop of any contacts”

Figure 2 shows our main results. It depicts the average planned defense effort as well as the support for governmental regulations by treatment group. We find—in comparison to the baseline treatment—that a Deontological moral appeal increases individually planned defense measures, while there is no clear effect of a Consequentialist moral appeal on individually planned defense measures. A Consequentialist moral appeal does not increase high-opportunity cost contact reduction, it only increases planned hand cleaning efforts, i.e. the low-opportunity cost option.

In Table 1, we provide regression results. Our preferred specification relies on OLS and includes covariates. OLS results without covariates correspond to Fig. 2. We also provide results of a Tobit specification, which considers potential left- and right-censoring of answers. We observe an additional reduction of 30.3% in the number of physical social contacts (*p* value = 0.043) for participants in the Deontological treatment and a reduction of 13.9% (*p* value = 0.348) for participants in the Consequentialist treatment. With regard to hand cleaning efforts, we find an additional increase by 41.0% (*p* value = 0.003) for participants in the Deontological treatment and an increase by 24.9% (*p* value = 0.069) for those in the Consequentialist treatment. We report *p* values corrected for multiple hypothesis testing in Table 8 in the “Appendix”, which shows that our results are qualitatively robust for the two approaches suggested by List et al. (2019). With regard to our first hypothesis, we therefore summarize:

**Table 1:** Moral appeals on private public good contributions and support for gov. regulation

	Planned contacts		Planned hand cleaning effort		Support for gov. reg.		Change cont. wrt. gov. reg.	
	(1)	(2)	(3)	(4)	(5)	(6)	(7)	(8)
*Model A: OLS estimator*								
Deont.	− 0.290*	− 0.303**	0.416***	0.410***	0.109***	0.110***	0.140***	0.132***
	(0.151)	(0.150)	(0.140)	(0.139)	(0.042)	(0.041)	(0.042)	(0.041)
Conseq.	− 0.141	− 0.139	0.289**	0.249*	0.107***	0.097**	0.100**	0.084**
	(0.149)	(0.148)	(0.139)	(0.137)	(0.041)	(0.040)	(0.041)	(0.041)
*Model B: Tobit estimator*								
Deont.	− 0.638**	− 0.667**	0.497***	0.487***	0.109***	0.110***	0.140***	0.132***
	(0.300)	(0.297)	(0.175)	(0.173)	(0.042)	(0.041)	(0.042)	(0.041)
Conseq.	− 0.300	− 0.299	0.345**	0.292*	0.107***	0.097**	0.100**	0.084**
	(0.291)	(0.288)	(0.172)	(0.170)	(0.041)	(0.040)	(0.041)	(0.040)
Covariates	No	Yes	No	Yes	No	Yes	No	Yes
Observations	3447	3430	3416	3399	3447	3430	3441	3424

Notes: Regression results based on OLS and Tobit estimators. The latter addresses concerns for left- and right-censoring of answers. The change in contacts and hand cleaning effort is measured with a 15-point log-scale as described in the main text and allows for an interpretation in percentage points. For the support for gov. regulations and the change in contacts wrt. gov. regulations we use their z-score to allow for an interpretation in units of standard deviations. Covariates include respondent’s age, gender, and categorical dummy variables for education and household income. Robust standard errors in parentheses. *$p<0.1$, **$p<0.05$, ***$p<0.01$

#### **Result 1**

*Moral appeals can increase planned private public good contributions. The Deontological appeal has an overall stronger effect compared to the Consequentialist appeal: It can induce planned actions not only with low but also with high opportunity costs.*

In terms of support for governmental regulation, we find that both moral appeals are about equally effective in increasing support for regulations. In particular, moral appeals increase the support for governmental regulations by 0.11 (Deontological treatment) and 0.10 (Consequentialist treatment) standard deviations. And similarly, participants report a larger reduction in contacts of 0.13 [0.09] standard deviations than required by regulations after being exposed to the Deontological [Consequentialist] appeal compared to the control treatment. We thus establish:

#### **Result 2**

*Both Deontological and Consequentialist moral appeals increase support for governmental actions by a similar magnitude.*

### Heterogeneous Treatment Effects

While extensions of the SIR model suggests tailored policy intervention to specific population groups (Acemoglu et al. 2020; Alfaro et al. 2020; Brotherhood et al. 2020; Gollier 2020; Grimm et al. 2020), we now focus on subgroups and examine which individuals show the strongest reaction to those moral appeals. Following from this motivation, we compare young with old respondents, and male with female in Table 4 in the Appendix. For the age split, we use a threshold of 60 years, which is motivated from the field of epidemiology and commonly differentiates between epidemiological high- and low-risk groups. As an alternative age-group classification, we also differentiate between respondents < 30, 30–60, and > 65 years in Table 5 in the “Appendix”. To rule out that differences between age groups are driven by different risk perceptions, we also control for the perceived risk to get ill if participants get infected.

We find that particularly respondents who are younger than 60 years show a stronger reaction to our moral appeals at almost every outcome. Respondents older than 60 years, instead, only show a modest effect on their planned hand cleaning effort. This also holds true for distinguishing respondents younger and older than 65 years. While the support for governmental regulations seems to be unaffected for men, we observe a higher support by treated women. Across these population groups, the treatment effect is on average higher in the Deontological treatment than the Consequentialist appeal, which is in line with our previous results. When we focus on respondents younger than 30 years, this pattern seems to change. In particular, the consequentialist appeal increases planned hand cleaning effort, while the deontological appeal increases governmental support. As we control for the perceived health risk of an infection, we do not expect differences to be driven by different health risk. We rather suspect that trust in government and life-experience could explain this effect and leave a systematic investigation of the underlying mechanisms for future research.

We further differentiate between individuals along with a measure of impure altruism. To do so, we rely on a survey question asking “As far as you reduce physical, social contacts or take protective efforts such as intensive hand washing, in what proportions (in percentage points that sum up to 100%) do you do this in order to (1) Protect yourself and members of your household [x%]; (2) Protect your family and close friends [y%]; Protect other people [100-x-y%].”. For our whole sample, the mean [median] weight put on (1) oneself is 50 [52]%, (2) on family and friends is 30 [30], and (3) on others is 18 [20]. In Table 6 in the “Appendix”, we provide subgroup analysis based on this altruism measure. While respondents with at least the average weight put on themselves show no reaction to the moral appeal, those with a higher weight put on their family, friends, and others plan to contribute much more to the public good than those who did not see a moral appeal. When we, in addition, only consider the weight put on persons not belonging to the group of family members and friends, we also find a stronger effect on the planned reduction of contacts in the Deontological treatment but not robustly in the Consequentialist treatment, and we in particular find no reaction with regard to governmental support. Hence, Deontological moral appeals seem particularly effective for more altruistic respondents and can lead to more pronounced private public good contributions.

## Conclusion

We have studied the effect of moral suasion on the private provision of public goods. While the use of moral interventions in environmental economics is gaining traction (e.g. Carlsson et al. 2019; Ito et al. 2018), little attention has been put on scrutinizing the ethical reflections underlying the moral statements and how they interact with regulations. We exploit the COVID-19 pandemic as an exemplary case where private contributions to public health are of critical importance to study the effect of moral appeals following the two major schools of Consequentialist and Deontological ethics. The spread of COVID-19 is a relevant case study for this, as it allows us to elicit treatment effects from the general population and as it imposes a natural experiment on the private public good provision under uncertainty, where individual actions impose severe externalities.

Overall, we find that moral appeals by a medical doctor treating COVID-19 patients trigger intended private public good contributions relative to a control treatment. This effect is stronger for an appeal to moral duty (following Deontological ethics) than for an appeal to the consequences of individual actions (following Consequentialist ethics). We observe this for both high-cost and low-cost public good contributions, which we measure via planned reduction in physical social contacts and hand cleaning effort, respectively. Finally, we find that both appeals seem equally effective in increasing the support for governmental regulations. We further uncover heterogeneous treatment effects, such as younger males being particularly susceptible to a Deontological appeal for reducing contacts, which should be further explored in future studies.

There are different potential explanations for our results. First, Deontological ethics might be more prominent and accepted within the German population, due to the pronounced intellectual tradition following the German philosopher Immanuel Kant. Consequentialist ethics, instead, are more widespread in Anglo-Saxon countries, such as the UK. Thus, the cultural setting of Germany could explain the stronger effect of the Deontological appeal as compared to the Consequentialist appeal. Second, a Consequentialist appeal makes thinking about outcomes but potentially also about individual opportunity costs in attaining certain outcomes more salient. This could explain why a Consequentialist appeal can increase planned actions with lower opportunity costs (hand washing), but fails to induce actions with higher opportunity costs (social distancing). From a Consequentialist viewpoint, individual action is less important after all—what matters is the societal outcomes, which may be best attained via governmental regulation. Finally, respondents being exposed to the Consequentialist appeal might have only considered the consequences of their own actions or underestimated the social costs. The Deontological appeal, instead, could have made a socially optimal norm more salient such that individuals act more in line with the social planner solution that internalizes all infection externalities.

In closing, we want to emphasize limitations of our study and outline fruitful avenues for future research. First, our results are based on planned behavior and we do not observe the actual private public good contributions of our respondents. This would require observing realized behavior employing a tracing app, for instance, which could not be implemented. Quaas et al. (2020) show, however, that stated reductions in contacts in the previous week are correlated with mobility reductions as captured by cell phone movements. Hence, it seems plausible that stated planned behavior is indicative of actual behavior.

Second, future research should examine to what extent effects of moral appeals according to specific schools of ethics are culture or country-specific, as the prominence of different schools of ethics varies across countries. For instance, we expect that Germans put a higher weight on Deontological moral arguments compared to UK citizens, and, accordingly, one could expect that a Deontological moral appeal has a stronger relative effect in Germany as compared to the UK.

Third, future research should carry out a more systematic exploration of heterogeneous treatment effects in an even larger sample and focus on the interaction with social norms. While we observe that the majority of respondents acts in line with regulations or does even more than required by regulation, social norms to follow rules might enable or even increase the effectiveness of moral appeals. Such an analysis could guide the way for tailoring moral suasion for specific sub-groups and thus make moral suasion a more potent policy tool aligning individual actions with societal objectives to overcome social dilemmas. Forth, trust in government or the moral sender might affect the effectiveness of moral appeals and differ between population groups. A recent study by Sabat et al. (2020), conducted after ours, shows that trust in information from the government in the COVID-19 context tends to correlate positively with age. We are cautious, however, in which direction trust interacts with our treatments. High levels of trust ex ante could pronounce the effect of moral appeals, but reactions by policymakers, for example, might also affect trust ex post. As we have not elicited trust in our survey, we leave the question of how trust interacts with the effect of different moral appeals on the support for governmental regulation for future research.

Fifth, the effect of moral appeals might depend on the specific private-public good character of the good under investigation. However, we do not find that moral appeals do less strongly or less significantly affect planned hand washing efforts relative to planned contacts (Table 6, Panel C and Panel D) for those individuals who put a relatively higher share on protecting others. Again we leave a more thorough investigation on how the effect of moral appeals differs with ratio of private to public benefits affects to future research.

Finally, and naturally, it will be interesting to study how the results we obtain on the likely effectiveness of moral appeals in the context of COVID-19 carry over to collective action problems related to environmental public goods. Welsch (2020), for instance, shows that self-reported moral values from the European Social Surveys are positively correlated with climate-friendly behavior and support of climate-friendly regulations. It is therefore promising to study the role of moral suasion on voluntary contributions to conserve biodiversity, such as via individual food choices, and to mitigate climate change, such as via individual transportation choices.

## Figures and Tables

**Fig. 4:**
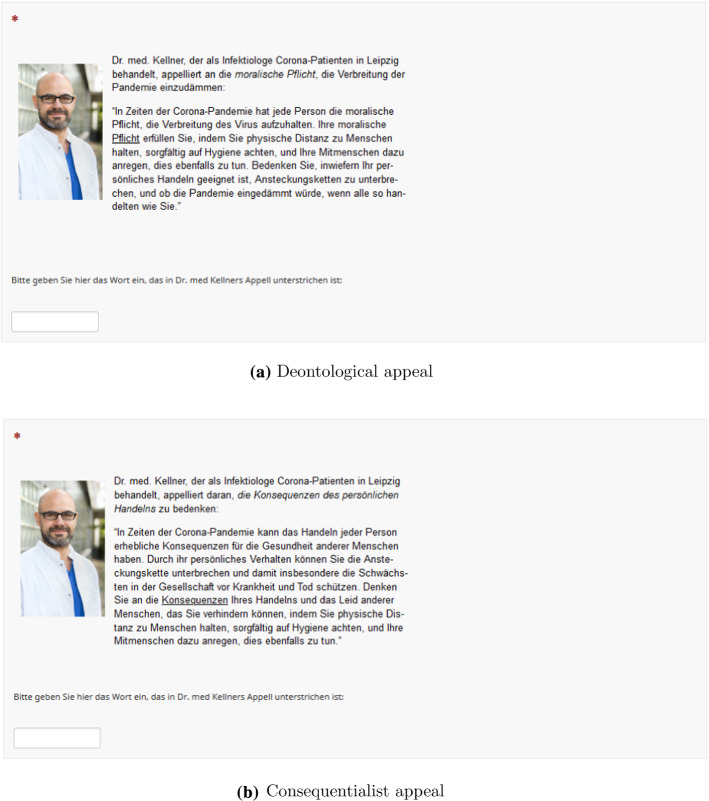
Screenshots of moral appeals. *Notes* Both figures illustrate how respondents saw the moral appeals. Respondents in the control group saw no statement

## Appendix

See Figs. 3 , 4 and Tables 2, 3, 4, 5, 6, 7, 8 and 9.

**Table 2:** Descriptive statistics of relevant survey responses

	All	Population group			
		Young men	Young women	Old men	Old women
Change in planned contacts	3.49	3.81***	3.14***	3.55	3.59
(15-point Likert scale)	(3.59)	(3.43)	(3.42)	(3.76)	(4.10)
Change in planned hand cleaning effort	11.46	10.92***	11.54	11.85**	12.06***
(15-point Likert scale)	(3.30)	(3.36)	(3.30)	(2.96)	(3.38)
*Support for gov. regulation (in %)*					
Are too much	0.09	0.12***	0.09	0.06**	0.05***
	(0.28)	(0.32)	(0.29)	(0.24)	(0.21)
Are appropriate	0.31	0.34**	0.29*	0.33	0.27*
	(0.46)	(0.47)	(0.45)	(0.47)	(0.44)
Are too little	0.60	0.54***	0.62	0.61	0.69***
	(0.49)	(0.50)	(0.49)	(0.49)	(0.46)
*Change wrt. regulation (in %)*					
Less than required	0.07	0.09***	0.06	0.07	0.03***
	(0.25)	(0.29)	(0.24)	(0.25)	(0.17)
According to regulations	0.30	0.34***	0.32*	0.21***	0.24**
	(0.46)	(0.47)	(0.47)	(0.41)	(0.43)
More than required	0.63	0.57***	0.62	0.72***	0.73***
	(0.48)	(0.50)	(0.49)	(0.45)	(0.44)
Observations	3,448	1118	1297	551	482

The table shows mean values and standard deviations in parentheses. Change in planned contacts and planned hand cleaning effort was elicited with a logarithmic Likert scale as described in the main text. Stars indicate the significance of the mean values to the average mean values of the other groups (t-tests). *$p<0.1$, **$p<0.05$, ***$p<0.01$

**Table 3:** Balance tests

	Control	Deont.	Conseq.
Age	49.12	50.44*	50.57*
	(15.38)	(15.30)	(15.40)
Female	0.52	0.51	0.52
	(0.50)	(0.50)	(0.50)
*Household income*			
< 1500	0.16	0.17	0.16
	(0.37)	(0.38)	(0.37)
1500–3000	0.40	0.41	0.41
	(0.49)	(0.49)	(0.49)
3000–4000	0.23	0.23	0.21
	(0.42)	(0.42)	(0.41)
$\ge$ 4000	0.20	0.19	0.22
	(0.40)	(0.40)	(0.41)
*Education*			
University degree	0.21	0.22	0.20
	(0.41)	(0.41)	(0.40)
A-levels / vocational training	0.20	0.18	0.19
	(0.40)	(0.38)	(0.39)
Secondary school	0.36	0.38	0.38
	(0.48)	(0.48)	(0.48)
Secondary general school	0.22	0.23	0.23
	(0.42)	(0.42)	(0.42)
No degree	0.00	0.00	0.00
	(0.07)	(0.07)	(0.06)
Observations	1109	1141	1198

Table shows mean values and standard deviations in parentheses. Stars indicate the significance in differences between the treatment groups and the control group (t-tests). *$p<0.1$, **$p<0.05$, ***$p<0.01$

**Table 4:** Moral appeals on private public good contributions and support for gov. regulation by subgroups

	Planned contacts		Planned hand cleaning effort		Support for gov. reg.		Change cont. wrt. gov. reg.	
	(1)	(2)	(3)	(4)	(5)	(6)	(7)	(8)
*Panel A: Young respondents (age* $<60$)								
Deont.	− 0.442**	− 0.421**	0.300*	0.299*	0.109**	0.107**	0.147***	0.131***
	(0.173)	(0.172)	(0.169)	(0.167)	(0.051)	(0.051)	(0.050)	(0.049)
Conseq.	− 0.263	− 0.241	0.219	0.196	0.082*	0.084*	0.077	0.060
	(0.172)	(0.171)	(0.166)	(0.164)	(0.049)	(0.049)	(0.049)	(0.050)
Observations	2414	2335	2396	2321	2415	2336	2408	2333
*Panel B: Old respondents (age* $\ge 60$)								
Deont.	0.067	0.024	0.599**	0.654***	0.083	0.055	0.091	0.090
	(0.303)	(0.303)	(0.249)	(0.252)	(0.072)	(0.071)	(0.075)	(0.074)
Conseq.	0.153	0.055	0.380	0.427*	0.139*	0.102	0.122*	0.108
	(0.296)	(0.299)	(0.251)	(0.256)	(0.071)	(0.071)	(0.074)	(0.073)
Observations	1033	1006	1020	993	1032	1005	1033	1006
*Panel C: Male respondents*								
Deont.	− 0.489**	− 0.475**	0.409**	0.380*	0.042	0.035	0.071	0.052
	(0.214)	(0.210)	(0.198)	(0.196)	(0.059)	(0.059)	(0.060)	(0.058)
Conseq.	− 0.195	− 0.170	0.115	0.061	0.081	0.069	0.114**	0.086
	(0.216)	(0.214)	(0.198)	(0.196)	(0.057)	(0.056)	(0.058)	(0.057)
Observations	1668	1663	1652	1647	1668	1663	1666	1661
*Panel D: Female respondents*								
Deont.	− 0.119	− 0.143	0.433**	0.452**	0.179***	0.186***	0.213***	0.209***
	(0.213)	(0.210)	(0.198)	(0.197)	(0.058)	(0.057)	(0.057)	(0.057)
Conseq.	− 0.101	− 0.107	0.458**	0.437**	0.133**	0.129**	0.090	0.079
	(0.206)	(0.202)	(0.193)	(0.192)	(0.058)	(0.057)	(0.058)	(0.057)
Observations	1779	1767	1764	1752	1779	1767	1775	1763
Covariates	No	Yes	No	Yes	No	Yes	No	Yes

OLS regressions. The change in contacts and hand cleaning effort is measured with a 15-point log-scale as described in the main text and allows for an interpretation in percentage points. For the support for gov. regulations and the change in contacts wrt. gov. regulations we use their z-score to allow for an interpretation in units of standard deviations. Covariates include respondent‘s age, gender, and categorical dummy variables for education and household income. In Panel A und B, we also control for the perceived probability to get ill. Robust standard errors in parentheses. *$p<0.1$, **$p<0.05$, ***$p<0.01$

**Table 5:** Moral appeals on private public good contributions and support for gov. regulation by subgroups

	Planned contacts		Planned hand cleaning effort		Support for gov. reg.		Change cont. wrt. gov. reg.	
	(1)	(2)	(3)	(4)	(5)	(6)	(7)	(8)
*Panel A: Age* < 30								
Deont.	− 0.107	− 0.034	0.525	0.546	0.242**	0.225**	0.132	0.058
	(0.394)	(0.377)	(0.391)	(0.401)	(0.111)	(0.114)	(0.114)	(0.117)
Conseq.	− 0.341	− 0.077	0.992***	0.968***	0.071	0.028	0.141	0.064
	(0.401)	(0.384)	(0.366)	(0.370)	(0.112)	(0.114)	(0.113)	(0.115)
Observations	426	406	422	402	426	406	425	405
*Panel B: Age 30–65*								
Deont.	− 0.435**	− 0.428**	0.335**	0.341**	0.109**	0.104**	0.149***	0.143***
	(0.178)	(0.180)	(0.170)	(0.169)	(0.051)	(0.051)	(0.050)	(0.049)
Conseq.	− 0.176	− 0.205	0.161	0.173	0.092*	0.098**	0.079	0.076
	(0.178)	(0.179)	(0.170)	(0.168)	(0.050)	(0.050)	(0.050)	(0.050)
Observations	2373	2304	2357	2292	2374	2305	2368	2303
*Panel C: Age* > 65								
Deont.	0.155	0.116	0.533*	0.657**	− 0.013	− 0.018	0.063	0.054
	(0.403)	(0.398)	(0.313)	(0.312)	(0.090)	(0.090)	(0.096)	(0.096)
Conseq.	0.097	0.010	0.139	0.241	0.121	0.091	0.078	0.053
	(0.377)	(0.381)	(0.312)	(0.316)	(0.084)	(0.085)	(0.092)	(0.092)
Observations	648	631	637	620	647	630	648	631
Covariates	No	Yes	No	Yes	No	Yes	No	Yes

OLS regressions. The change in contacts and hand cleaning effort is measured with a 15-point log-scale as described in the main text and allows for an interpretation in percentage points. For the support for gov. regulations and the change in contacts wrt. gov. regulations we use their z-score to allow for an interpretation in units of standard deviations. Covariates include respondent‘s age, gender, categorical dummy variables for education and household income, and the perceived probability to get ill. Robust standard errors in parentheses. *$p<0.1$, **$p<0.05$, ***$p<0.01$

**Table 6:** Moral appeals on private public good contributions and support for gov. regulation by subgroups

	Planned contacts		Planned hand cleaning effort		Support for gov. reg.		Change cont. wrt. gov. reg.	
	(1)	(2)	(3)	(4)	(5)	(6)	(7)	(8)
*Panel A: Low level of altruism (Share to protect me* $>50$)								
Deont.	− 0.005	− 0.040	0.095	0.056	0.080	0.074	0.127*	0.117
	(0.253)	(0.249)	(0.245)	(0.244)	(0.074)	(0.073)	(0.073)	(0.071)
Conseq.	0.032	− 0.000	− 0.141	− 0.205	0.137*	0.113	0.188**	0.157**
	(0.250)	(0.248)	(0.246)	(0.245)	(0.072)	(0.071)	(0.073)	(0.072)
Observations	1145	1139	1136	1130	1146	1140	1143	1137
*Panel B: Median level of altruism (Share to protect me* $=50$)								
Deont.	− 0.179	− 0.202	0.177	0.217	0.147*	0.159*	0.119	0.128
	(0.314)	(0.315)	(0.271)	(0.270)	(0.084)	(0.084)	(0.082)	(0.081)
Conseq.	0.017	0.048	0.033	0.009	0.121	0.122	0.072	0.061
	(0.308)	(0.305)	(0.267)	(0.263)	(0.083)	(0.083)	(0.083)	(0.082)
Observations	865	861	857	853	864	860	864	860
*Panel C: High level of altruism (Share to protect me* $<50$)								
Deont.	− 0.630***	− 0.679***	0.826***	0.822***	0.128**	0.127**	0.173***	0.163**
	(0.235)	(0.232)	(0.220)	(0.219)	(0.063)	(0.062)	(0.065)	(0.063)
Conseq.	− 0.398*	− 0.422*	0.776***	0.756***	0.080	0.069	0.054	0.047
	(0.233)	(0.232)	(0.215)	(0.214)	(0.061)	(0.060)	(0.062)	(0.061)
Observations	1435	1428	1421	1414	1435	1428	1432	1425
*Panel D: High level of altruism (Share to protect others* $>20$)								
Deont.	− 0.770***	− 0.822***	0.480*	0.485*	0.075	0.073	0.051	0.039
	(0.269)	(0.266)	(0.254)	(0.253)	(0.074)	(0.073)	(0.074)	(0.073)
Conseq.	− 0.120	− 0.108	0.406*	0.357	0.086	0.073	0.025	0.011
	(0.279)	(0.276)	(0.244)	(0.242)	(0.071)	(0.070)	(0.072)	(0.071)
Observations	1076	1069	1063	1056	1076	1069	1073	1066
Covariates	No	Yes	No	Yes	No	Yes	No	Yes

OLS regressions. We measure altruism through the survey question “As far as you reduce physical, social contacts or take protective efforts such as intensive hand washing, in what proportions (in percentage points that sum up to 100%) do you do this in order to (1) Protect yourself and members of your household [x%]; (2) Protect your family and close friends [y%]; Protect other people [100-x-y%].”. The change in contacts and hand cleaning effort is measured with a 15-point log-scale as described in the main text and allows for an interpretation in percentage points. For the support for gov. regulations and the change in contacts wrt. gov. regulations we use their z-score to allow for an interpretation in units of standard deviations. Covariates include respondent‘s age, gender, and categorical dummy variables for education and household income. Robust standard errors in parentheses. *$p<0.1$; **$p< 0.05$; ***$p < 0.01$

**Table 7:** Robustness checks

	Preferred model	Controling for contact ban	Including slow and fast respondents	Including invalid answers	Reweighted control groups	
	(1)	(2)	(3)	(4)	(5)	(6)
*Outcome A: Planned contacts*						
Deont.	− 0.301**	− 0.301**	− 0.359**	− 0.281*	− 0.296*	
	(0.150)	(0.150)	(0.148)	(0.149)	(0.151)	
Conseq.	− 0.140	− 0.141	− 0.203	− 0.078		− 0.124
	(0.148)	(0.148)	(0.147)	(0.148)		(0.149)
Observations	3433	3433	3531	3485	2242	2295
*Outcome B: Hand cleaning effort*						
Deont.	0.410***	0.407***	0.395***	0.362***	0.410***	
	(0.139)	(0.139)	(0.137)	(0.139)	(0.140)	
Conseq.	0.250*	0.242*	0.265*	0.223		0.254*
	(0.137)	(0.137)	(0.136)	(0.137)		(0.138)
Observations	3402	3402	3501	3453	2226	2270
*Outcome C: Support for gov. regulation*						
Deont.	0.109***	0.105**	0.104**	0.104**	0.105**	
	(0.041)	(0.041)	(0.041)	(0.041)	(0.041)	
Conseq.	0.098**	0.087**	0.096**	0.091**		0.093**
	(0.040)	(0.040)	(0.040)	(0.040)		(0.040)
Observations	3433	3433	3532	3485	2242	2295
*Outcome D: Change in contacts wrt. gov. regulation*						
Deont.	0.132***	0.129***	0.124***	0.127***	0.131***	
	(0.041)	(0.041)	(0.040)	(0.041)	(0.041)	
Conseq.	0.084**	0.078*	0.084**	0.078*		0.079*
	(0.041)	(0.040)	(0.040)	(0.040)		(0.041)
Observations	3427	3427	3526	33479	2238	2292
Covariates	Yes	Yes	Yes	Yes	Yes	Yes
Only valid answer	Yes	Yes	Yes	No	Yes	Yes
Contact ban dummy	No	Yes	No	No	No	No
With slow & fast resp.	No	No	Yes	No	No	No
Weighted control group	No	No	No	No	Yes	Yes

OLS regressions. The change in contacts and hand cleaning effort is measured with a 15-point log-scale as described in the main text and allows for an interpretation in percentage points. For the support for gov. regulations and the change in contacts wrt. gov. regulations we use their z-score to allow for an interpretation in units of standard deviations. In column (2), we include a dummy variable equal to one of respondents participated after the German contact ban has been announced on March 22, 2020. In column (3), we include respondents that took less [more] than 3 [60] min to complete the survey. In column (4), we include respondents that failed to enter the right underlined word in the statement with the moral appeal. And finally, in columns (5) and (6), we reweight the control group against each treatment group using entropoy balancing (Hainmueller 2012) to address potential concerns about the randomization into treatment groups. Covariates include respondent‘s age, gender, and categorical dummy variables for education and household income. Robust standard errors in parentheses. *$p<0.1$, **$p<0.05$, ***$p<0.01$

**Table 8:** Multiple hypothesis testing

Outcome	Treatment	DI	*p*-values	
			Unadj.	Multiplicity adj.
Planned contacts	Deont.	0.290	0.056**	0.098*
Planned contacts	Conseq.	0.141	0.367	0.367
Hand cleaning effort	Deont.	0.416	0.004***	0.028**
Hand cleaning effort	Conseq.	0.289	0.032**	0.087*
Support for gov. reg.	Deont.	0.109	0.014**	0.068*
Support for gov. reg.	Conseq.	0.107	0.008***	0.043**
Change contacts wrt. gov. reg	Deont.	0.140	0.000***	0.000***
Change contacts wrt. gov. reg	Conseq.	0.100	0.015**	0.058*

Table compares the result for different multiple hypothesis testing procedures. DI refers to difference in means. The multiplicity adjusted *p* values are based on List et al. (2019). *$p<0.1$, **$p<0.05$, ***$p<0.01$

**Table 9:** Relevant survey questions

Variable name	Question and answer options
Planned contacts	Compared to the same week last year, by what percentage will you reduce or increase your physical, social contacts in the coming week? *(1) reduction to one-tenth, (2) ..., (3) halving, (4) ..., (5) reduction by 10%, (6) ..., (7) reduction by 1%, (8) unchanged, (9) increasing by 1%, (10) ..., (11) increasing by 10%, (12) ..., (13) doubling, (14) ..., (15) tenfold increase*
Planned hand cleaning effort	Compared to the same week last year, by what percentage will you reduce or increase your intensive hand washing (longer than 20 s) in the coming week? *(1) reduction to one-tenth, (2) ..., (3) halving, (4) ..., (5) reduction by 10%, (6) ..., (7) reduction by 1%, (8) unchanged, (9) increasing by 1%, (10) ..., (11) increasing by 10%, (12) ..., (13) doubling, (14) ..., (15) tenfold increase*
Support for governmental regulation	The current government measures to contain the corona pandemic...*(1) are way too much, (2) ..., (3) are too much, (4) ..., (5) ..., (6) are appropriate, (7) ..., (8), (9), are too little, (10) ..., (11) are way too little*
Change in contacts wrt. gov. regulation	Relative to the governmental regulations, I will limit my physical, social contacts as follows: *(1) participation at Corona-parties, (2) ..., (3) much less than required, (4) ..., (5) ..., (6) according to the regulations, (7) ..., (8) ..., (9) much more than required, (10) ..., (11) complete stop of any contacts*

For the full questionnaire, please refer to our Pre-Analysis Plan available at https://doi.org/10.1257/rct.5573-1.1

## Footnotes

1 Recent field experiments, for example, study the impact of moral suasion payment behavior (Bursztyn et al. 2019; Pruckner and Sausgruber 2013), tax evasion (Bott et al. 2020), sovereign bond purchases by domestic banks (Ongena et al. 2019), and book returns at a public library (Apesteguia et al. 2013).

2 Goldstein et al. (2008, p. 473), for instance, study how normative appeals impact towel reuse by hotel guests, by means of the following environmental message “HELP SAVE THE ENVIRONMENT. You can show your respect for nature and help save the environment by reusing your towels during your stay.”. Ito et al. (2018, p. 248) study how a moral suasion treatment affects energy consumption by informing energy costumers about the importance of energy conservation in critical peak-demand hours; Their moral plea does not relate closely to a specific theory of ethics: “substantial energy conservation will be required for the society in ‘critical peak-demand hours’ on summer and winter peak-demand days, in which electricity supply will be very limited relative to demand.”

3 Examples include, for instance, the role of intrinsic values in environmental valuation (Johansson-Stenman 1998) and sustainability approaches in the presence of uncertainty (Howarth 1995). In a recent review on how welfare economics has been extended and enriched by philosophical departures beyond utilitarianism, Fleurbaey (2019) finds that economics has hitherto hardly incorporated Deontological or contractual ethics.

4 We pre-registered the survey at the AEA RCT Registry (10.1257/rct.5573-1.1), have made all data and code for replication available at the Harvard Dataverse (10.7910/DVN/WEIWDK), and provide further details on the study in the “Appendix”. The survey respondents are representative for the German population in terms of gender, age, education, and income. We excluded 112 respondents that answered the survey in less [more] than 3 [60] min due to concerns regarding fast-clicking or inattention. We also excluded another 53 respondents that entered an incorrect word in a treatment check (see below). In Table 7, we show that our results are robust against these choices and would lead to marginally lower effect sizes.

5 We provide balance tests between control and treatment groups in Table 3. We find no significant differences in respondents’ characteristics at the 5% level. Yet, respondents in both treatment groups are significantly older at the 10% significance level. We show in Table 7 that our results hold for weighted samples where we reweight the control group to match the characteristics of each treatment group using entropy balancing (Hainmueller 2012).

6 In particular, we asked participants to complete the statement “The current government measures to contain the corona pandemic ...” with answer options ranging from “going way too far” to “are not nearly enough” and “Relative to the governmental regulations, I will limit my physical, social contacts as follows:” with answer options ranging from “participation in Corona-parties” to “complete avoidance of all contacts”.

7 In Table 7, we include a dummy variable for the contact ban and show that our results still hold once we control for changes in the regulation.

8 Both hypotheses have been pre-registered with the survey as described above. Besides these two hypotheses, the pre-analysis plan also includes a hypothesis on the effect of the moral treatment on truth-telling as measured by a coin-tossing task. As the latter hypothesis is out of the scope of this paper, we omit this hypothesis and re-number the two remaining relevant hypotheses compared to the pre-analysis plan.

## References

[CR1] Acemoglu D, Chernozhukov V, Werning I, Whinston MD (2020) A multi-risk SIR model with optimally targeted lockdown. Working Paper 27102, National Bureau of Economic Research, Cambridge

[CR2] Alfaro L, Faia E, Lamersdorf N, Saidi F (2020) Social interactions in pandemics: fear, altruism, and reciprocity. Working paper 27134, National Bureau of Economic Research, Cambridge

[CR3] Andreoni J (1990). Impure altruism and donations to public goods: a theory of warm-glow giving. Econ J.

[CR4] Andreoni J (2007). Giving gifts to groups: how altruism depends on the number of recipients. J Public Econ.

[CR5] Apesteguia J, Funk P, Iriberri N (2013). Promoting rule compliance in daily-life: evidence from a randomized field experiment in the public libraries of Barcelona. Eur Econ Rev.

[CR6] Bénabou R, Falk A, Tirole J (2018) Eliciting moral preferences, Unpublished manuscript. https://scholar.princeton.edu/sites/default/files/rbenabou/files/moral_preferences_august_9_snd.pdf

[CR7] Bott KM, Cappelen AW, Sørensen EØ, Tungodden B (2020). You’ve got mail: a randomized field experiment on tax evasion. Manag Sci.

[CR8] Brotherhood L, Kircher P, Santos C, Tertilt M (2020) An economic model of the covid-19 epidemic: the importance of testing and age-specific policies. Discussion paper 14695, Centre for Economic Policy Research, London

[CR9] Bursztyn L, Fiorin S, Gottlieb D, Kanz M (2019). Moral incentives in credit card debt repayment: evidence from a field experiment. J Polit Econ.

[CR10] Carlsson F, Gravert C, Johansson-Stenman O, Kurz V et al (2019) Nudging as an environmental policy instrument. Working paper 756, Department of Economics, University of Gothenburg

[CR11] Dal Bó E, Dal Bó P (2014). “Do the right thing:” the effects of moral suasion on cooperation. J Public Econ.

[CR12] Dannenberg A (2015). Leading by example versus leading by words in voluntary contribution experiments. Soc Choice Welf.

[CR13] Daube M, Ulph D (2016). Moral behaviour, altruism and environmental policy. Environ Resour Econ.

[CR14] Egebark J, Ekström M (2016). Can indifference make the world greener?. J Environ Econ Manag.

[CR15] Everett JAC, Colombatto C, Chituc V, Brady WJ, Crockett M (2020) The effectiveness of moral messages on public health behavioral intentions during the COVID-19 pandemic, Unpublished manuscript. https://osf.io/9yqs8

[CR16] Farboodi M, Jarosch G, Shimer R (2020) Internal and external effects of social distancing in a pandemic. Working paper 27059, National Bureau of Economic Research, Cambridge

[CR17] Fleurbaey M (2019). Economic theories of justice. Ann Rev Econ.

[CR18] Gerlagh R (2020) Closed-form solutions for optimal social distancing in a sir model of COVID-19 suppression. Working paper No. 8335, CESifo, Munich

[CR19] Goldstein NJ, Cialdini RB, Griskevicius V (2008). A room with a viewpoint: using social norms to motivate environmental conservation in hotels. J Consum Res.

[CR20] Gollier C (2020) Cost-benefit analysis of age-specific deconfinement strategies. Unpublished manuscript. https://drive.google.com/file/d/1Hs7VBjQC9OWn1a_vEyaTExf97uORKBId/view

[CR21] Grimm V, Mengel F, Schmidt M, (2020) Extensions of the SEIR model for the analysis of tailored social distancing and tracing approaches to cope with COVID-19. Unpublished manuscript. http://www.wirtschaftstheorie.wiso.uni-erlangen.de/wp-content/uploads/2020/04/Grimm_Mengel_Schmidt__2020.pdf 10.1038/s41598-021-83540-2 PMC7893058 33603113

[CR22] Hainmueller J (2012). Entropy balancing for causal effects: a multivariate reweighting method to produce balanced samples in observational studies. Polit Anal.

[CR23] Harsanyi JC (1953). Cardinal utility in welfare economics and in the theory of risk-taking. J Polit Econ.

[CR24] Howarth RB (1995). Sustainability under uncertainty: a deontological approach. Land Econ.

[CR25] Ito K, Ida T, Tanaka M (2018). Moral suasion and economic incentives: field experimental evidence from energy demand. Am Econ J Econ Policy.

[CR26] Johansson-Stenman O (1998). The importance of ethics in environmental economics with a focus on existence values. Environ Resour Econ.

[CR27] Kallbekken S, Sælen H (2013). ‘Nudging‘ hotel guests to reduce food waste as a win–win environmental measure. Econ Lett.

[CR28] List JA, Shaikh AM, Xu Y (2019). Multiple hypothesis testing in experimental economics. Exp Econ.

[CR29] Maskin E (1978). A theorem on utilitarianism. Rev Econ Stud.

[CR30] Mill JS (1859) Utilitarianism. Liberty, Representative Government

[CR31] Nyborg K, Anderies JM, Dannenberg A, Lindahl T, Schill C, Schlüter M, Adger WN, Arrow KJ, Barrett S, Carpenter S, Chapin FS, Crépin A-S, Daily G, Ehrlich P, Folke C, Jager W, Kautsky N, Levin SA, Madsen OJ, Polasky S, Scheffer M, Walker B, Weber EU, Wilen J, Xepapadeas A, de Zeeuw A (2016). Social norms as solutions. Science.

[CR32] Ongena S, Popov A, Van Horen N (2019). The invisible hand of the government: moral suasion during the European sovereign debt crisis. Am Econ J Macroecon.

[CR33] Pruckner GJ, Sausgruber R (2013). Honesty on the streets: a field study on newspaper purchasing. J Eur Econ Assoc.

[CR34] Quaas M, Meya J, Schenk H, Bos B, Drupp M, Requate T (2020) The social cost of contacts: theory and evidence for the COVID-19 pandemic in germany. CESifo working paper no. 8347. https://www.cesifo.org/DocDL/cesifo1_wp8347.pdf 10.1371/journal.pone.0248288 PMC7978372 33740007

[CR35] Roemer J (2019). How we cooperate: a theory of Kantian optimization.

[CR36] Romans JT (1966). Moral suasion as an instrument of economic policy. Am Econ Rev.

[CR37] Sabat I, Neuman-Böhme S, Varghese NE, Barros PP, Brouwer W, van Exel J, Schreyögg J, Stargardt T (2020). United but divided: policy responses and people’s perceptions in the EU during the COVID-19 outbreak. Health Policy.

[CR38] van Doremalen N, Bushmaker T, Morris DH, Holbrook MG, Gamble A, Williamson BN, Tamin A, Harcourt JL, Thornburg NJ, Gerber SI, Lloyd-Smith JO, de Wit E, Munster VJ (2020). Aerosol and surface stability of sars-cov-2 as compared with sars-cov-1. N Engl J Med.

[CR39] Welsch H (2020). Moral foundations and voluntary public good provision: the case of climate change. Ecol Econ.

[CR40] Young HP (2015). The evolution of social norms. Ann Rev Econ.

